# Invited Frontiers Commentary. Tier Climbing Article: Redefining Neuromarketing as an Integrated Science of Influence

**DOI:** 10.3389/fnins.2017.00022

**Published:** 2017-01-26

**Authors:** Sven Braeutigam

**Affiliations:** Department of Psychiatry, Oxford Centre for Human Brain Activity, University of OxfordOxford, UK

**Keywords:** neuromarketing, influence, scaling, reductionism, ethics

For a long time, traders and scholars alike have attempted to unravel and understand the mechanisms underlying the human behaviors of selling and consuming goods and services. Recently, technological advances have given rise to the field of neuromarketing, which enhances traditional marketing research through the use of neuroscience technologies in order to better understand consumer preferences and choice processes, as well as to inform the design and presentation of products (Ambler et al., 2004; Ariely and Berns, 2010). Despite many advances, however, neuromarketing is still fragmented, produces predominantly correlational results and lacks a strong theoretical framework, but this situation is going to change.

The Applied Neuromarketing Consortium at Northwestern University, led by Prof. Breiter, recently published a ground-breaking theoretical paper suggesting that, in order to advance neuromarketing as a science, one has to embrace a broader perspective on what the authors call influence. Influence is considered to be the balance between preferences within an individual or group that affect the outside world, and external preferences that affect the individual or group. As such, influence is more than just shifting the distribution of choices to one favored by a corporation, government or other entity (Breiter et al., 2015). Influence involves, so the argument runs, many mental operations measurable to some extent at both the behavioral and neuronal level. Thus, influence is thought of as being present across multiple systems and spatio-temporal levels of measurement, from group measures to measures of individual behavior to neural groups.

This approach comprises many domains of sciences requiring, as the authors rightly state, as yet not fully evolved theoretical schemes for how relevant cognitive processes such as perception and attention interact. So, does that mean influence as defined here is too difficult to quantify to be useful? Certainly not! Breiter and colleagues argue that a feature known as scaling (or scale invariance) can be used to study influence across multiple scales of behavior. Scaling is a feature of laws or objects that do not change if scales of time, length, or other parameters are dilated or contracted by a common factor. Scaling is an important theoretical concept and has been shown to hold to a good approximation in natural systems (e.g., Freyer et al., 2012). For example, the logarithmic spiral describes the flight path of falcons (Tucker et al., 2000), shape of broccoli cabbage, and wind patterns in cyclones (Figure 1, left).

**Figure 1:**
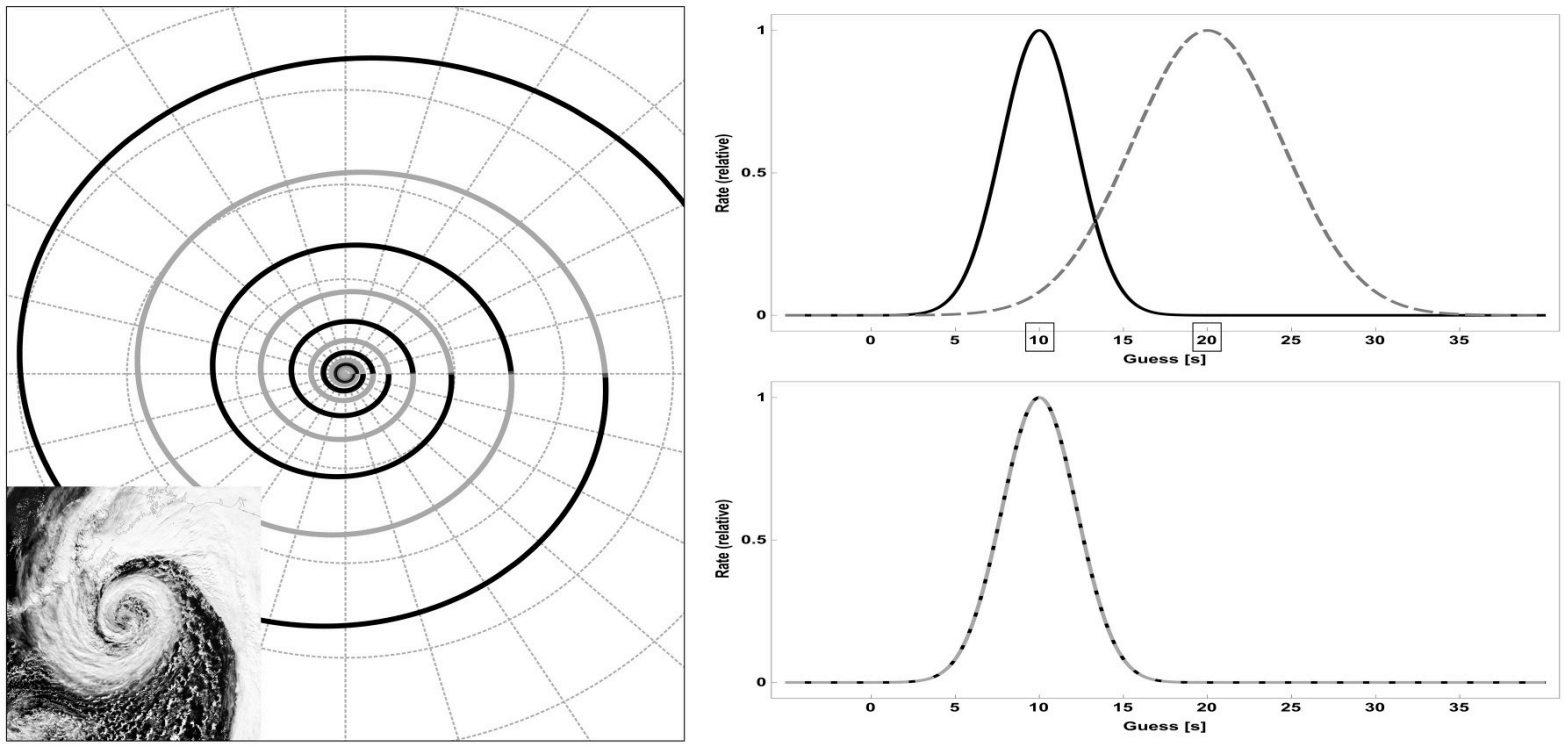
**(Left)** The logarithmic spiral is a mathematical curve that is scale-invariant. The precise definition of invariance in this case is beyond the scope of this paper, but, loosely speaking, the overall shape of the curve repeats itself on each turn (black and gray line segments). In other words, after each turn, the curve looks exactly the same just at a greater magnification. Inset: an extratropical cyclone over Alaska exhibiting a large and approximately scale-invariant spiral pattern (image adapted from http://earthobservatory.nasa.gov/NaturalHazards/view.php?id=79300). **(Right)** Scale invariance in human behavior. When people have to judge elapsed time, their guesses closely follow a normal distribution peaking around the actual time. Critically, the variance of distribution is proportional to the length of time interval (upper panel, solid: 10 s interval, dotted: 20 s). This implies scale invariance, i.e., response functions become equal under time dilation and/or contraction (lower panel, dotted curve represents a time -> time/2 transformation; real data can be found in Buhusia and Oprisan, 2013).

In humans, scale invariance is observed in interval timing, where errors in time estimation scale up linearly with the estimated duration. In other words, relative errors depend on the subject or group but not the interval itself (Buhusia and Oprisan, 2013; see also Savage and West, 2006). At the level of large communities and societies, scaling laws have been shown to exist for patterns of human travel (Brockmann et al., 2006). Moreover, scaling may exist not only along a single dimension but also across different levels of spatio-temporal organization within one organism or even between species. For example, although humans and rats have opposite rest/activity cycles, the heartbeat fluctuations in both species exhibit similar power-law (a form of scale invariance) correlations over a range of time scales linked to the endogenous circadian rhythms (Hu et al., 2008). At the behavioral level, the authors' own experimental work suggest that approach and avoidance behaviors may scale from individual to group behavior, and possibly to neuronal scales (Kim et al., 2010).

While scaling cannot be expected to provide a tight links between neuronal activity and aggregate economic behavior any time soon, the approach is powerful and has great promise to yield a better understanding of how the different levels of human behavior interact and possibly necessitate each other. Clearly, Breiter and colleagues aim for an ambitious goal requiring time consuming work. Nevertheless, there appear several domains in which their work could have an impact soon.

From a conceptual perspective, one would expect that the study of influence can make a significant contribution to the broader debate about reductionism in neuroscience. In particular in the applied fields of neuromarketing and organizational cognitive neuroscience, scholars have voiced concerns about how insights gained at the individual neuronal level can inform complex interactions occurring at various levels, from small groups of individuals, to large companies, to societies (Lindebaum and Zundel, 2013). An insight into the putative scaling laws underlying mental function will not solve all of the deeply philosophical aspects of reductionism. Such insight, however, has the potential to clarify the extent to which high-level human behavior is an emergent feature as opposed to be inherent in the low-level properties (Blomberg, 1994).

From an ethical perspective, one may assume that the work by Breiter and colleagues will encourage increased utilization of brain-imaging technologies in commercial, organizational, and governmental settings. This could fuel concerns neuroscience based approaches might be used in ways that infringe personal privacy to an unacceptable degree. It seems more likely, however, that a better understanding of the broad mechanisms underlying influence will inform guidelines, recommendations and regulations aimed at the protection of individual autonomy, averting harm, and exploitation caused by the research. At current, there is no conclusive evidence that neuroscience based technology permits the types of manipulations that critics envisage (Murphy et al., 2008; Fisher et al., 2010), but the future is difficult to predict and one has to observe the development and decide if and when regulatory interventions are needed.

From an application perspective, insights into the broader aspects of influence could help to better understand the long-term outcomes and/or consequences of marketing communication efforts. For example, the food industry as well as the consumer appear too much intent on the value of eating and drinking without considering possible long-term outcomes, such as eating related health issues, which, in turn, can change behavior at many scales (Schultz, 2015) in the long term. Along these lines, it is conceivable that the influence framework will have clinical applications, in particular for conditions involving large (time) scales, such as addiction and decisions making in aging.

Whatever future developments will bring, an integrated approach to influence seems a brilliant concept—Neuromarketing 2.0 is on the horizon!

## Author contributions

The author confirms being the sole contributor of this work and approved it for publication.

### Conflict of interest statement

The author declares that the research was conducted in the absence of any commercial or financial relationships that could be construed as a potential conflict of interest.
